# BRAZILIAN PERSPECTIVES AND TRENDS FOR MINIMALLY INVASIVE SURGERY IN THE TREATMENT OF HALLUX VALGUS

**DOI:** 10.1590/1413-785220253304e289483

**Published:** 2025-09-08

**Authors:** Elizabeth de Alvarenga Borges da Fonseca, Caio de Almeida Oliveira, Guilherme Bottino Martins, Lourenço Galizia Heitzmann, Gustavo Demasi Quadros de Macedo, Wellington Farias Molina

**Affiliations:** 1 Hospital Servidor Público Estadual de São Paulo, Departamento de Ortopedia e Traumatologia, São Paulo, SP, Brazil.

**Keywords:** Hallux Valgus, Diffusion of Innovation, Professional Training, Hallux Valgus, Difusão de Inovações, Capacitação Profissional

## Abstract

**Objective:**

To evaluate trends in corrective surgery for hallux valgus in Brazil, focusing on minimally invasive techniques, regardless of generation.

**Methods:**

Information was obtained through a questionnaire, using the "Survey" tool, to foot and ankle orthopedists; conducted by the Orthopedics and Traumatology Service of IAMSPE. Outcomes were measured and evaluated through tables and incidence. Data from 54 orthopedic doctors were evaluated, regardless of the surgical technique used to correct hallux valgus.

**Results:**

A trend toward preference for the minimally invasive technique was observed, due to better clinical outcomes and early rehabilitation.

**Conclusion:**

There is a need for further study and in-depth analysis of the minimally invasive technique for the treatment of hallux valgus, considering that this has become the main method of choice for the treatment of this condition. ***Level of Evidence IV; Case Series***.

## RESUMO

**Objetivo:**

Avaliar as tendências da cirurgia corretiva para o hálux valgo no Brasil, com foco nas técnicas minimamente invasivas, independentemente da geração.

**Métodos:**

As informações foram obtidas por meio de um questionário, utilizando a ferramenta "Survey", a ortopedistas de Pé e tornozelo; realizada pelo Serviço de Ortopedia e Traumatologia do IAMSPE. Os desfechos foram mensurados e avaliados por meio de tabelas e incidência. Foram avaliados dados de 54 médicos ortopedistas, independentemente, da técnica cirúrgica utilizada para correção do hallux valgo.

**Resultados:**

Observou-se uma tendência de preferência pela técnica minimamente invasiva, devido aos melhores desfechos clínicos e à reabilitação precoce.

**Conclusão:**

Há necessidade de estudo e aprofundamento da técnica minimamente invasiva para o tratamento do hálux valgo, considerando que esta vem se tornando o principal método de escolha para o tratamento dessa condição. ***Nível de Evidência IV; Série de Casos***.

## INTRODUCTION

The definition of Hallux Valgus (HV) was proposed by Carl Hueterem in 1871, as the lateral (valgus) deviation of the hallux, accompanied by a medial deviation from the head of the first metatarsus.^1^

HV is the most common pathology of hallux, its etiology is multifactorial, having its origin in the most varied reasons, from genetic factors to associated systemic diseases.^2^ This change is often bilateral and affects mainly women in adulthood due to the presence of extrinsic factors mainly the use of inappropriate shoes.^3^

Conservative treatment should always be considered, especially for pain control and maintenance of the "functionality" of the limb, especially in cases without prior follow-up or in early stages. They are part of the non-surgical treatment: optimization of footwear with the decrease of heels height (up to 4 centimeters) and the enlargement of the anterior chamber; physiotherapy stimulating the strengthening of intrinsic musculature of the foot, and also, the use of palms to support the longitudinal arc and discharge in the central metatarsals.^4^

In situations where non-surgical methods are not effective and when we have severe deformity, surgical treatment is indicated. Several surgical techniques have been developed to try to solve the problem.^5-7^

Minimally invasive techniques are evolving and are gaining ground in all areas of orthopedic surgery. With the benefits of reduced time and surgical aggression, stress to the patient and better recovery time.^8,9^

Percutaneous surgery (PS) of the foot, began to establish itself in Spain and France over the last two decades, modifying various concepts and creating techniques. Although the first works developed in the United States of America (USA) were more than 50 years ago, the PS foot did not have immediate interest due to the lack of theoretical and practical foundations, as well as the scarcity of scientific works developed and published.^10^

The first method of minimally invasive surgery to treat hallux valgus was described by Isham et al.^11^ and involved the use of a percutaneous drill to perform an osteotomy in a medial closure cone without the use of fixing material.

Other techniques were developed over time and percutaneous surgery was spread in clinical practice and in scientific publications.

In Brazil the number of publications on the technique has been increasing, which demonstrates its growth in popularity. However, in relation to the practice of foot and ankle surgeons what are the perspectives and trends for these techniques?

## MATERIALS AND METHODS

The study was approved by the Ethics Committee under number 79497524.0.0000.5463. An opinion poll of the type "Survey" was made available by a digital platform for evaluation by foot and ankle surgeons orthopedists, titled by the Brazilian Association of Foot and ankle, to answer the questionnaire.

The questionnaire was elaborated in "google forms" and the survey was made available in the first quarter of 2024 - consisting of 14 questions about the treatment and follow-up of hallux valgus by the minimally invasive technique.

Fifty-four experts participated in obtaining the data – the completion of the Survey was voluntary and the results remained anonymous.

The definition of the minimally invasive technique for the treatment of hallux valgus and the proposal for this research were informed to the participants; even if another surgical technique was used this was not a reason for exclusion from the research and only differentiation; any doubt about the project was resolved via e-mail.

## RESULTS

**Figure 1:**
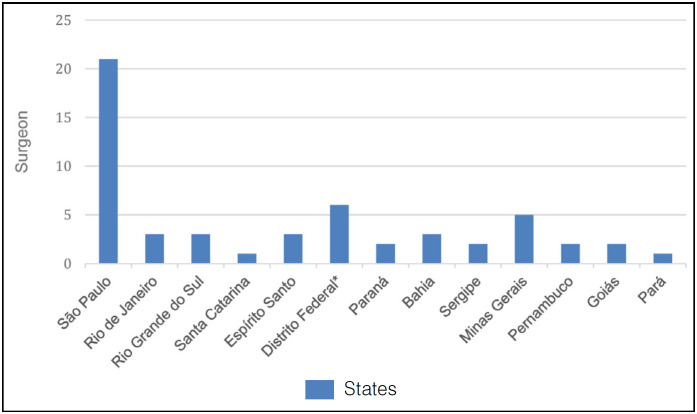
Geographical distribution of participants.

The survey had a multicenter aspect, as it was answered by 54 foot and ankle surgeons in Brazil, distributed in 12 states and all regions of Brazil (São Paulo, Rio de Janeiro, Rio Grande do Sul, Espírito Santo, Paraná, Bahia, Sergipe, Minas Gerais, Pernambuco, Goiás, Santa Catarina and Pará), plus the Federal District. São Paulo was the state with the highest number of responses (21 out of 54 or 38.8%). (Figure 1)

**Figure 2:**
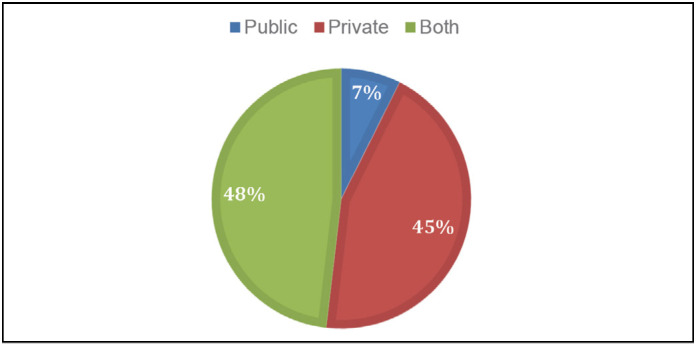
Type of participant performance service.

A large proportion of surgeons work in mixed healthcare systems (26 out of 54), others only in private services (24 out of 54) and a minority exclusively in public services (4 out of 54) (Figure 2). However, 55,6% claim to work in School Hospital.

**Figure 3:**
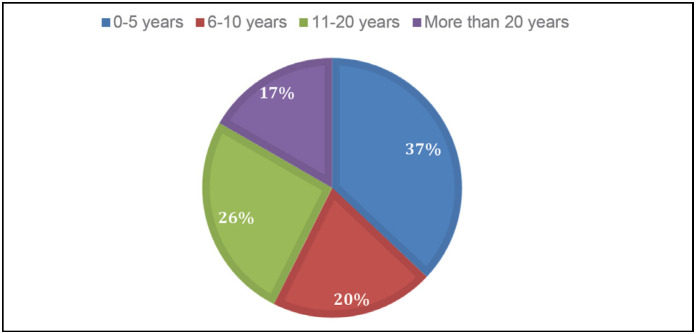
Training time in specialization in foot and ankle.

The training time in medicine and foot and ankle surgery of professionals was heterogeneous, with the majority in the last 5 years (Figure 3). Despite young training in foot and ankle surgery, 59.2% of participants claim to have learned the techniques through extracurricular courses.

**Figure 4:**
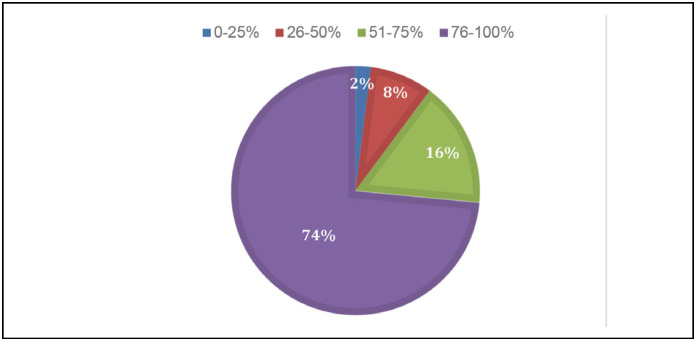
Proportion of cases in which the surgeon performs the minimally invasive surgery for the treatment of hallux valgus.

Of the 54 participating surgeons, the vast majority (90.74% or 49 out of 54) know the minimally invasive methods for hallux valgus surgery and perform them; the rest do not apply the technique in their daily life. Of these, 73.5% perform minimally invasive surgery in 75-100% of their cases, showing the current preference for minimally invasive surgery. (Figure 4)

Severe hallux deformity was the major contraindication for the procedure (reported by 9 out of 14 patients who noted some restrictions, with the main concern being the hallux itself). Other contraindications mentioned include underlying comorbidities, instability of the first tarsometatarsal joint, among other restrictions.

**Figure 5:**
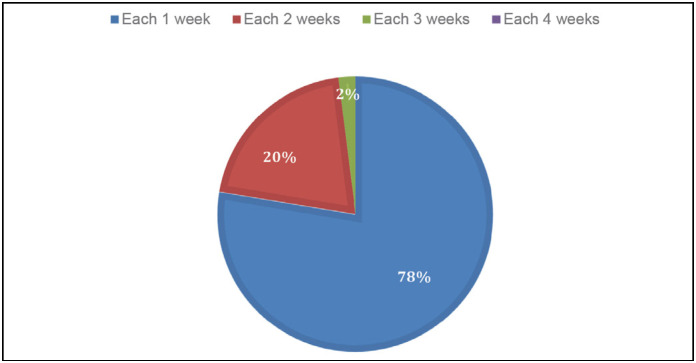
Time for the curative exchange.

After the minimally invasive procedure has been completed satisfactorily, almost all surgeons release the load immediately (93.8%) with appropriate sandals, other surgeons release in 2 weeks and only every 2 weeks. The change of curative in 1 week was preferred by the majority (77.6%). (Figure 5)

**Figure 6:**
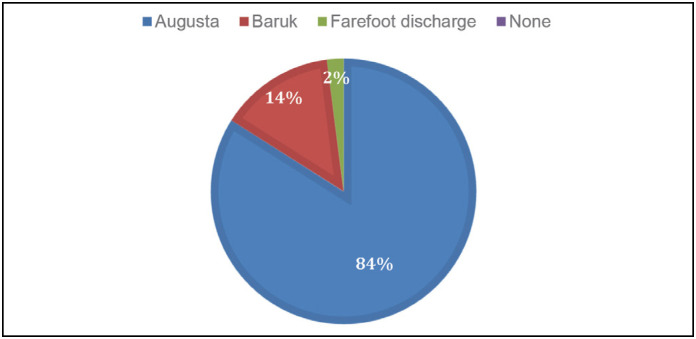
Type of postoperative sandal.

The indication of the use of the postoperative sandals is carried out by all surgeons, preferably by the type of *Augusta*. (Figure 6)

## DISCUSSION

According to Nogueira,^12^ the technological diffusion is considered very dynamic with the incorporation of innovations, and abandonments of techniques and practices considered obsolete. Thus, the minimally invasive approaches of hallux valgus are considered a technological innovation in the phase of diffusion and incorporation. The results of the research on the adoption of minimally invasive surgery to surgically treat hallux valgus in Brazil reflect this spread in clinical practice.

Of the 54 surgeons involved, 90.74% know and use the technique in their practice. This reflects a significant acceptance of percutaneous surgery for the surgical treatment of hallux valgus in all regions of Brazil, even in the northern region of the country where there are relatively few foot and ankle surgeons, although our research cannot prove this statistically due to the small number of participants in the region.

Severe hallux valgus deformity was identified as the primary contraindication by 64.29% of surgeons reporting some restriction to the use of the technique according to our study. Other mentioned restrictions include associated comorbidities and instability of the tarsometatarsal joint. This finding is in accordance with the study of Tan and Thevendran,^10^ where the severity of the deformity is also pointed out as the main contraindication for the indication of percutaneous surgeries in the Asia-Pacific region. Similarly, the European study by Maffulli et al.^13^ highlights that although percutaneous surgery is promising, there are limitations due to the heterogeneity of cases and the lack of conclusive evidence about its effectiveness in severe deformities.

The learning curve involves the completion of several cases until you reach the necessary proficiency, in addition to appropriate training in both specialization and extracurricular courses. The importance of rigorous training and continuous practice to overcome the learning curve is emphasized in the courses conducted by Brazilian surgeons. This search for the best technique offers substantial benefits in terms of patient recovery and satisfaction.^7,14^

After performing the minimally invasive surgery, most surgeons release the load immediately with the use of appropriate sandals. This postoperative protocol suggests confidence in the stability of percutaneous fixation performed in Brazil.^5,15,16^ In contrast, in Asia-Pacific, most surgeons allow full load between four and six weeks after surgery, suggesting a more conservative approach to postoperative recovery.^10^

The clinical results obtained with percutaneous procedures for the correction of mild to moderate deformities are comparable to those obtained with other percutaneous osteotomies of the distal metatarsus and with most series of open surgical procedures.^17,18^ Thus, percutaneous surgery presents advantages in the postoperative period as an immediate burden with the use of appropriate sandals and due to the reduced aggression of soft parts.

The growing popularity of minimally invasive surgery highlights the need for more comparative research between traditional open surgeries, especially in terms of medium and long-term results, especially in the detection of patient satisfaction.^7^

## CONCLUSION

We concluded that the adoption of percutaneous surgery for correction of hallux valgus in Brazil is broad and well received, with positive postoperative results and rehabilitation protocols that favor the rapid recovery of patients, such as early load.

However, the severity of the deformity and other comorbidities continue to be significant challenges that limit the use of the technique in some cases. The comparison with data from Asia-Pacific and Europe provides a global context that reinforces the relevance of Brazilian findings and suggests that, despite regional differences, the trends and challenges of minimally invasive surgery are similar internationally.
